# Two Languages and One Aphasia: A Systematic Scoping Review of Primary Progressive Aphasia in Chinese Bilingual Speakers, and Implications for Diagnosis and Clinical Care

**DOI:** 10.3390/brainsci16010020

**Published:** 2025-12-24

**Authors:** Weifeng Han, Lin Zhou, Juan Lu, Shane Pill

**Affiliations:** College of Education, Psychology and Social Work, Flinders University, Bedford Park, SA 5042, Australia; lin.zhou@flinders.edu.au (L.Z.);

**Keywords:** primary progressive aphasia, Chinese bilingual speakers, tonal phonology, logographic writing, cross-linguistic assessment, culturally responsive clinical practice

## Abstract

**Background/Objectives**: Primary progressive aphasia (PPA) is characterised by progressive decline in language and communication. However, existing diagnostic frameworks and assessment tools are largely based on Indo-European languages, which limits their applicability to Chinese bilingual speakers whose linguistic profiles differ markedly in tonal phonology, logographic writing, and bilingual organisation. This review aimed to (a) describe how PPA presents in Chinese bilingual speakers, (b) evaluate how well current speech–language and neuropsychological assessments capture these impairments, and (c) identify linguistically and culturally informed strategies to improve clinical practice. **Methods**: A systematic review was conducted in accordance with the PRISMA-ScR guidelines. Four databases (PubMed, Scopus, Web of Science, PsycINFO) were searched, complemented by backward and forward citation chaining. Eight empirical studies met the inclusion criteria. Data were extracted on participant characteristics, PPA variant, language background, speech–language and writing profiles, and assessment tools used. Thematic analysis was applied to address the research questions. **Results**: Across variants, Chinese bilingual speakers demonstrated universal PPA features expressed through language-specific pathways. Mandarin speakers exhibited tone-segment integration errors, tonal substitution, and disruptions in logographic writing. Lexical-semantic degradation reflected homophony and compounding characteristics. Bilingual individuals showed parallel or asymmetric decline influenced by dominance and usage. Standard English-based naming, repetition, and writing assessments did not reliably capture tone accuracy, radical-level writing errors, or bilingual patterns. Sociocultural factors, including stigma, delayed help-seeking, and family-centred care expectations, further shaped diagnostic pathways. **Conclusions**: Chinese PPA cannot be meaningfully assessed using tools designed for Indo-European languages. Findings highlight the need for tone-sensitive repetition tasks, logographic writing assessments, bilingual diagnostic protocols, and culturally responsive communication-partner support. This review provides a comprehensive synthesis to date on Chinese bilingual PPA and establishes a foundation for linguistically inclusive diagnostic and clinical models.

## 1. Introduction

Primary progressive aphasia (PPA) is a neurodegenerative syndrome characterised by the progressive decline of language functions resulting from frontotemporal or Alzheimer’s disease pathology [1]. Despite substantial advancements in the PPA literature, research remains heavily concentrated on English and other Indo-European languages, e.g., [2,3,4], limiting our understanding of how PPA presents in speakers of structurally distinct languages [5]. We address this gap by focusing on Chinese bilingual speakers, a linguistically diverse and globally significant group [6] for whom PPA remains under-recognised and under-diagnosed.

Chinese languages differ from Indo-European languages in ways that may influence how PPA symptoms manifest and progress. Tonal phonology requires pitch contrasts to distinguish lexical meaning, which introduces vulnerabilities in tone production and perception that are rarely captured by non-tonal assessments. Evidence from Mandarin-speaking individuals with PPA demonstrates impairments in tone recognition, tone production, and tone-segment integration [7]. Morphological structure is predominantly compounding, with largely homophonous monosyllabic morphemes, suggesting that lexical-semantic decline may follow patterns different from those observed in English. The logographic writing system introduces additional orthographic demands, as character processing relies on visually complex combinations of strokes and radicals. This system is particularly vulnerable in PPA, with studies reporting radical substitution, stroke-order disruption, and visually similar character intrusions [8]. These structural features highlight why PPA in Chinese speakers cannot simply be inferred from the Indo-European evidence base. Although Chinese languages encompass substantial dialectal and typological diversity, including Mandarin, Cantonese, and Wu varieties, it is important to note that the empirical evidence currently available is heavily weighted toward Mandarin-speaking populations. Accordingly, while the review acknowledges broader Sinitic diversity as conceptually relevant, the synthesis and analysis primarily reflect findings from Mandarin-speaking individuals.

Bilingualism adds further complexity. Many Chinese speakers across Asia and the global diaspora use Mandarin or Cantonese (among many other Chinese dialects) alongside English or other languages, where a variety of factors impact Chinese learners’ English language acquisition process [9,10,11]. Research on cognitive ageing suggests that bilingualism may modify neural reserve and influence the trajectory of neurodegenerative disease [12]. In PPA specifically, bilingual individuals may show either parallel deterioration across their languages or asymmetric decline driven by proficiency, exposure, and linguistic distance [13]. Such findings indicate that bilingual language organisation interacts with neurodegenerative pathology in ways that are critical for understanding PPA in Chinese-background communities.

It should be pointed out that, in this review, the term bilingual is used as an umbrella descriptor encompassing heterogeneous language profiles. These include monolingual speakers of a single Chinese variety, intra-Sinitic bilinguals who use two or more Chinese varieties (e.g., Mandarin and Cantonese), and cross-family bilinguals who use a Chinese language alongside a non-tonal language such as English. Importantly, these groups differ in tonal structure, phonological overlap, script use, and patterns of language dominance, all of which may influence how PPA manifests and is assessed. Therefore, bilingualism is conceptualised with reference to established models of bilingual lexical access and language dominance, recognising that relative proficiency, usage patterns, and cross-language interaction may influence how neurodegenerative language decline manifests. However, given the limited and heterogeneous nature of the available studies, variant-specific effects of bilingualism are interpreted cautiously and framed as areas for future investigation rather than definitive conclusions.

Despite these linguistic and cognitive considerations, the diagnostic pathways available to Chinese-speaking individuals remain limited. Many naming, memory, and cognitive tasks commonly used with Chinese speakers were originally developed for English, which, as a result, makes them culturally and linguistically mismatched. Evidence from older Chinese adults, for example, demonstrates that the Boston Naming Test, one of the most widely used naming assessments, may not reliably differentiate semantic impairment from cultural unfamiliarity or lexical frequency differences when applied to Chinese-speaking populations [14]. Broader neuropsychological findings further suggest that applying non-normed Western tools to Chinese-background individuals increases the risk of diagnostic uncertainty, inflation of impairment scores, and potential misclassification [15]. Sociocultural factors further compound these issues. Stigma surrounding dementia, family-based help-seeking norms, and limited awareness of neurodegenerative conditions contribute to delays in assessment and reduced engagement in clinical services among Chinese-speaking communities [16]. These barriers are particularly consequential in PPA, where early identification is essential for planning and support.

As a response, the empirical studies synthesised in this review provide crucial insights into these challenges. They describe tonal and phonological impairments in Mandarin-speaking individuals with nfvPPA (the non-fluent/agrammatic variant) and lvPPA (the logopenic variant) [7,8], and semantic deterioration in svPPA (the semantic variant) [17]; as well as bilingual patterns of linguistic decline in Chinese-English speakers [13,18], psycholinguistic predictors of naming accuracy [19], and diagnostic differentiation using Chinese-language neurocognitive batteries [14,20]. Together, these studies offer a comprehensive picture to date of how PPA affects Chinese bilingual speakers and illustrate why linguistically and culturally informed approaches are necessary.

In this review, we build on the above findings by integrating evidence from linguistics, bilingualism, neuropsychology, and CALD healthcare to generate a more clinically meaningful understanding of PPA in Chinese-speaking populations. We address three research questions: RQ1—What speech, language, and writing features characterise Chinese bilingual speakers with PPA, and how do tone, script, and bilingualism shape these profiles across variants? RQ2—How effective are existing speech–language and neuropsychological assessment tools for identifying PPA in Chinese bilingual speakers, and what linguistic or cultural limitations reduce diagnostic accuracy? RQ3—What culturally and linguistically informed strategies can improve the diagnosis, communication support, and clinical care of Chinese bilingual individuals living with PPA?

Together, the three research questions form an integrated framework that directly reflects the focus of this review. RQ1 addresses what happens to speech, language, and writing in Chinese and Chinese-English speakers with PPA, therefore identifying how universal PPA features are expressed through tonal phonology, logographic script, and bilingual language organisation. RQ2 builds on these findings by examining how well existing speech–language and neuropsychological assessment tools capture these language-specific manifestations and highlights diagnostic blind spots and sources of misclassification. RQ3 then extends this synthesis to clinical practice and considers what can be carried out to improve assessment, diagnosis, and communication support through linguistically and culturally responsive approaches. Through answering the questions guiding this review, we aim to clarify what happens to speech and language in Chinese-speaking individuals with PPA and to identify evidence-informed directions for improving diagnostic accuracy and clinical support. By synthesising emerging empirical findings, we contribute to developing more linguistically inclusive models of PPA and enhancing clinical practice in culturally diverse societies.

## 2. Methods

We conducted a systematic scoping review to synthesise emerging evidence on primary progressive aphasia in Chinese bilingual speakers. A systematic design was appropriate because the literature in this area is heterogeneous, methodologically diverse, and limited in volume [21]. Although systematic search and screening procedures were applied, the review was designed to map and synthesise an emerging and heterogeneous evidence base rather than to estimate effect sizes or formally compare interventions. Terminology has therefore been standardised throughout to reflect a scoping review methodology. Our aim, therefore, was to map the breadth of empirical findings relevant to the research questions, identify key themes, and interpret the evidence through linguistically and culturally informed frameworks. We followed the PRISMA-ScR [22] reporting guidelines to maximise transparency and replicability.

### 2.1. Eligibility Criteria

We developed inclusion and exclusion criteria using a PICO-informed framework [23] tailored to the review purpose. PICO elements were applied iteratively to guide inclusion and data extraction rather than as rigid exclusion criteria. This approach allowed the review to capture a small but methodologically diverse evidence base while maintaining alignment with the research questions. Specifically, studies were eligible when they examined bilingual individuals diagnosed with PPA who spoke a Chinese language (e.g., Mandarin, Cantonese, Shanghainese, Hokkien, etc.). Only empirical studies reporting primary data on speech, language, writing, cognitive-linguistic performance, or diagnostic assessment were included. We excluded reviews, opinion papers, intervention protocols without primary data, or studies focusing on dementia or aphasia that did not meet diagnostic criteria for PPA. For clarity, we also excluded intervention studies targeting acquired (e.g., post-stroke) aphasia when PPA was not the index diagnosis, even if participants were Chinese-English bilinguals. Table 1 and Table 2 outline the PICO framework and inclusion and exclusion criteria that guided the searching and screening stages.

### 2.2. Information Sources and Search Strategy

We searched four databases, PubMed, Scopus, Web of Science, and PsycINFO, using adapted combinations of terms for PPA and Chinese languages. Search strings included terms related to PPA variants (e.g., “semantic variant”, “logopenic variant”, “nonfluent/agrammatic variant”), Chinese languages (e.g., “Mandarin”, “Wu”, “Cantonese”), and linguistic constructs relevant to speech and writing. We supplemented database searches with backward and forward citation chaining to identify additional eligible studies. We applied no date limits to maximise sensitivity, given the anticipated small size of the evidence base.

Search strategies combined controlled vocabulary terms and free-text keywords related to primary progressive aphasia, Chinese languages, bilingualism, and speech–language impairment. Boolean operators (AND/OR) were used to combine concepts. The final search was conducted on 13 October 2025. Searches were limited to peer-reviewed journal articles published in English; Chinese-language databases and grey literature were not systematically searched, reflecting the scope of this review and the peer-reviewed focus of the Special Issue.

### 2.3. Screening and Study Selection

**Figure 1:**
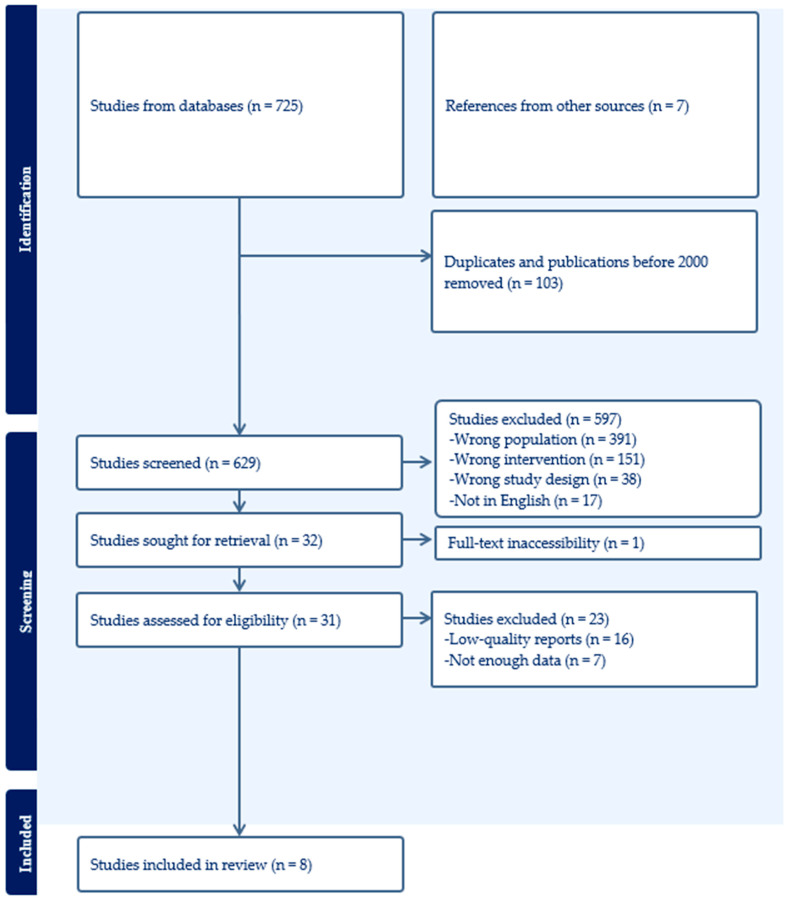
PRISMA flowchart.

All records were imported into Covidence [24] for de-duplication, followed by title and abstract screening. Titles and abstracts were screened independently by two authors, followed by full-text review. Discrepancies were resolved through discussion, with adjudication by a third author where consensus could not be reached. Given the scoping nature of the review, interrater agreement was established through iterative consensus-building rather than formal reliability statistics. Eight studies met all eligibility criteria and were included for analysis. The PRISMA flowchart in Figure 1 has the details of the identification and screening stages.

### 2.4. Data Extraction

We developed a structured extraction template capturing study design, participant characteristics, language background, PPA subtype, speech–language and writing features, assessment tools used, and key findings relevant to RQ1–RQ3. Extraction was conducted manually to ensure accuracy in interpreting Chinese-language terminology, tonal descriptions, orthographic features, and bilingual language histories. In addition to dual independent screening, study characteristics and thematic relevance were independently verified by a third author who had not conducted the initial data extraction for those studies. This verification focused on the accuracy of extracted study characteristics (Table 3) and the alignment of each study with the predefined research questions. Given the predominance of case studies, case series, and observational designs, a purpose-fit methodological appraisal approach was adopted rather than a generic risk-of-bias tool. Each study was evaluated for relevance to at least one research question, clarity of PPA variant characterisation, and sufficiency of linguistic or assessment detail to support thematic synthesis. Any discrepancies were resolved through discussion until a consensus was reached. The extracted data were then used to populate the study characteristics mapped to the research questions.

Further, in line with PRISMA guidance and the exploratory aims of this review, a purpose-fit methodological quality appraisal was conducted. Given that the included studies primarily comprised case studies, case series, and observational diagnostic investigations, formal tools designed for randomised controlled trials were not applied. Instead, studies were appraised using structured criteria relevant to their design and contribution to the research questions. These criteria included clarity of participant selection and diagnostic characterisation, adequacy and validity of linguistic or neuropsychological data, transparency of analytical procedures, and reporting quality. Appraisal was conducted independently by two authors and verified by a third author, with discrepancies resolved through discussion (see Table 4).

### 2.5. The Analytical Approach

Given the heterogeneity of study designs, populations, and outcome measures, we conducted a thematic synthesis rather than a quantitative synthesis [25]. Initial coding was conducted independently by two authors, focusing on speech–language features, assessment approaches, and clinical implications. Codes were compared and refined through discussion, and higher-order themes were developed iteratively. Theme interpretation was triangulated among authors to ensure coherence and consistency with the research questions. Throughout the process, our analysis was organised around the three research questions. For RQ1, we examined patterns of speech, language, and writing impairment and interpreted these with respect to tonal phonology, logographic orthography, and bilingual experience. For RQ2, we evaluated the assessment tools used in the included studies, drawing on CALD and Chinese neuropsychological literature to interpret diagnostic strengths and limitations. For RQ3, we integrated findings from the included studies with contextual evidence to propose linguistically and culturally informed clinical strategies.

## 3. Results

Given the heterogeneity of study designs and participant profiles, results are synthesised thematically in relation to the research questions rather than by study design. We synthesised findings from eight empirical studies that examined primary progressive aphasia in Chinese bilingual speakers. These studies varied in design, sample size, language background, and PPA subtype, but collectively offered converging insights relevant to our research questions. Table 3 provides an overview of study characteristics. The results are organised corresponding to RQ1 (core speech–language findings), RQ2 (assessment/diagnostic tools), and RQ3 (bilingual implications). Consistent with a systematic-review approach, we summarise patterns across studies while integrating contextual linguistic and neuropsychological evidence, where doing so clarifies mechanisms or strengthens interpretation [26]. As evidenced in Table 4, studies demonstrated clear diagnostic characterisation and adequate reporting of linguistic data, though variability was noted in sample size and depth of longitudinal evidence.

### 3.1. Speech and Language Impairment Profiles

Findings reported in this section are derived directly from the eight included studies; references to broader linguistic or sociocultural phenomena are used to contextualise these findings rather than to introduce independent empirical claims. Across the included studies, Chinese bilingual speakers with PPA demonstrated impairment patterns that aligned broadly with established PPA variants yet diverged from Indo-European profiles in ways shaped by tone, logographic writing, and bilingual experience. These distinctions were most evident in tonal production and perception, semantic-lexical degradation, phonological retrieval, orthographic processing, and cross-linguistic patterns of decline.

Mandarin-speaking individuals with nfvPPA and lvPPA exhibited clear tone-related difficulties. Tee, Deleon [7] reported impaired tone-segment integration, inaccurate tone production, and reduced tonal contrastiveness. These impairments occurred alongside motor-speech features such as slowed rate and articulatory groping. Similar disruptions in lexical tone were noted in lvPPA, which corroborates that phonological deficits in Chinese speakers extend beyond segmental processing to incorporate suprasegmental tone [27]. Such findings are also consistent with observations that tone is especially vulnerable in neurodegenerative conditions, where pitch-meaning mapping is compromised in ways not easily detected by non-tonal assessment tools [28].

Semantic variant PPA (svPPA) in Mandarin speakers was characterised by impaired confrontation naming, reduced semantic associations, and difficulty understanding low-frequency items [17]. Semantic errors reflected broader degradation of lexical concepts rather than simple retrieval failure. Mandarin’s extensive homophony [29] and compounding [30] may influence the trajectory of semantic decline, potentially accelerating confusion between phonologically identical morphemes.

Phonological deficits were prominent in logopenic variant (lvPPA) cases. Weekes (2020) [13] described pronounced word-finding difficulties, reduced repetition accuracy, and phonological paraphasias in a Chinese-English bilingual speaker with lvPPA, with impairment evident in both languages, though with differing severity. Filley et al. (2006) [18] similarly reported parallel deterioration in a Chinese-English bilingual individual. Jebahi et al. (2024) [19] further demonstrated that phonological complexity and lexical properties significantly predicted naming performance in Mandarin-speaking individuals across PPA variants. These findings highlight the central role of phonological processing in Chinese lvPPA, compounded by bilingual language organisation.

Logographic writing deficits were also notable in lvPPA. Tee, Lorinda Kwan-Chen [8], for example, documented radical substitutions, mislocated strokes, visually similar character intrusions, and structural simplifications. These errors illustrate the vulnerability of orthographic-semantic pathways in PPA when applied to logographic script, which reflects both phonological and visuoconstructive deterioration. Logographic error patterns cannot be meaningfully compared to alphabetic spelling errors, therefore further emphasising the need for Chinese-specific diagnostic frameworks.

Chinese-English bilinguals showed variability in the relative degree of impairment across languages. Weekes (2020) [13] observed asymmetrical deterioration favouring the historically dominant language, whereas Filley et al. (2006) [18] described a more parallel decline. These patterns underscore the influence of proficiency, dominance, and language environment on bilingual PPA trajectories. Bilingualism research suggests that neural reserve and language-use patterns may modulate decline in multilingual speakers [12], which helps explain individual variability observed in the reviewed studies. It is important to note, however, that observations regarding parallel or asymmetric decline are based on a small number of bilingual case studies. As such, these patterns should be interpreted as illustrative rather than generalisable, which further highlights the need for systematic investigation in larger bilingual cohorts.

Overall, findings across all eight studies indicate that PPA in Chinese speakers manifests through mechanisms that reflect universal PPA features but are shaped by Chinese tonal phonology, homophony-driven lexical architecture, and logographic orthography. These patterns are interpreted in light of established properties of Chinese languages, which may help explain the empirical observations reported in the included studies. Bilingual speakers further exhibit cross-linguistic patterns that complicate diagnostic interpretation.

### 3.2. Assessment and Diagnostic Challenges

The included studies illustrated substantial diagnostic limitations when Western-based assessment tools are applied to Chinese-speaking individuals with PPA. These challenges arose from linguistic mismatch, cultural unfamiliarity, limited Chinese-language norms, and the absence of tone- and radical-specific tasks in standard batteries.

Multiple studies highlighted the limitations of naming tasks. For example, Li et al. (2023) [14] showed that the Boston Naming Test, even when adapted for Mandarin, retains cultural and lexical biases that reduce discriminatory power. Chao et al. (2013) [17] demonstrated that semantic degradation in svPPA cannot always be separated from cultural unfamiliarity when semantic categories do not align with Chinese cultural knowledge. Differences in lexical frequency and homophony may also affect naming patterns in ways not accounted for in Indo-European norms.

Repetition tasks, widely used to diagnose lvPPA, are often calibrated to stress-timed, non-tonal languages. Tee, Deleon [7], for example, showed that Mandarin speakers produced tonal errors that would not be captured by standard repetition scoring in Western tools. Kuo et al. (2023) [20] also noted that phonological complexity in Mandarin interacts with cognitive load during repetition, complicating classification across PPA variants.

Standard Western batteries also lack measures of logographic processing. For example, while character-writing errors offer diagnostic value in identifying lvPPA, no validated logographic writing battery exists for PPA assessment in Chinese speakers [8]. This gap limits clinicians’ ability to differentiate logographic deficits driven by phonological versus visuoconstructive pathways.

A lack of bilingual norms is also of primary concern. For example, bilingual participants in Weekes’ (2020) [13] and Filley et al.’s (2006) [18] studies were assessed using tools with no bilingual norms. As a result, patterns of asymmetric decline were difficult to interpret. Without measures of language dominance, proficiency history, and bilingual language entropy, i.e., balance between the languages used; see [31], standard assessments cannot reliably distinguish pathological decline from baseline bilingual variation. Contextual evidence from culturally and linguistically diverse (CALD) neuropsychology further indicates that misclassification risk is heightened when tools are not adapted linguistically [15].

To consolidate RQ1 and RQ2 findings and highlight diagnostic gaps, Table 5 summarises core speech–language features alongside the tools used and their limitations.

### 3.3. Sociocultural and Clinical Implications

Although sociocultural factors are not the primary focus of the included empirical studies, they are discussed here to contextualise how linguistic impairments may intersect with real-world clinical pathways. Sociocultural factors emerged across the evidence as critical influences on diagnosis, care access, and communication support. Although the primary studies did not focus explicitly on sociocultural determinants, their findings intersected with the broader CALD literature, therefore, allowing us to interpret Chinese PPA profiles within real-world clinical contexts.

Chinese-speaking individuals often delay help-seeking due to stigma surrounding dementia, reluctance to disclose communication difficulties, and culturally grounded expectations of family-managed care [16]. These patterns contribute to later presentation, reduced opportunity for early intervention, and increased diagnostic uncertainty when impairment is advanced at first contact. In bilingual contexts, family members often have differing language preferences or expectations, complicating communication assessment and support planning. Bilingual speakers may switch languages strategically or revert to earlier-acquired varieties, as noted in Weekes [13], which can be misinterpreted as pathological decline without appropriate bilingual assessment protocols.

Chinese-speaking families frequently serve as primary caregivers. Evidence suggests that caregiver stress and communication breakdowns are exacerbated in PPA, where language decline is the central symptom [32]. The findings on tone, repetition, and lexical retrieval difficulties suggest that everyday communication tasks, such as understanding tonal contrasts or identifying homophonous words, may be disproportionately affected in Chinese-speaking families. These challenges underscore the importance of communication-partner training, bilingual education resources, and culturally responsive counselling.

The evidence, therefore, indicates that diagnostic accuracy and care quality for Chinese bilingual speakers with PPA are shaped not only by linguistic and cognitive mechanisms but also by cultural norms, help-seeking behaviours, bilingual family dynamics, and systemic assessment limitations.

## 4. Discussion

We aimed to address three research questions concerning the nature of speech–language impairments (RQ1), the effectiveness of diagnostic tools (RQ2), and strategies to improve assessment and clinical care (RQ3). Across all three domains, the findings highlight both the universality of PPA and its language-specific manifestations in Chinese-speaking populations. They also demonstrate the need for culturally and linguistically responsive diagnostic and therapeutic approaches.

### 4.1. What Happens to Speech and Language in Chinese Bilingual Speakers with PPA? (RQ1)

The findings demonstrate that PPA in Chinese speakers reflects the core diagnostic triad of semantic, phonological, and motor speech impairments described in established PPA frameworks [33]. However, the manifestation of these impairments diverges systematically from Indo-European patterns due to the features of Chinese languages and, where relevant, bilingual organisation. Importantly, however, where structural linguistic properties of Chinese languages are discussed below, these are presented as theoretical frameworks to interpret the empirical findings from the included studies rather than as independent empirical evidence.

Tone emerged as a key vulnerability in both nfvPPA and lvPPA. The observed impairments in tone-segment integration, tonal contrastiveness, and tonal production accuracy [7] indicate that PPA disrupts suprasegmental-lexical mapping in ways specific to tonal languages. In Chinese, tone carries lexical meaning, making tonal deficits functionally equivalent to phonemic substitution errors in alphabetic languages, but with more pervasive communicative consequences. These findings extend prior evidence that tone processing deteriorates in neurodegenerative conditions and emphasise the importance of including tonal analysis in PPA assessment.

Lexical-semantic decline in svPPA also showed language-specific features. Chinese speakers demonstrated degradation of lexical concepts, difficulty retrieving low-frequency words, and breakdowns in semantic associations [17]. Given Chinese’s high degree of homophony and heavy reliance on compounding, semantic loss may progress in patterns distinct from those documented in English, where morphological and phonological cues differ substantially. This aligns with the existing literature, e.g., [34] that semantic deterioration in Chinese dementia often clusters around overlapping homophone sets, a pattern that may reflect greater lexical ambiguity. It is, however, important to note that conclusions regarding svPPA in Chinese speakers are based on very limited empirical evidence and should be interpreted cautiously, highlighting the need for further variant-specific research.

Phonological deficits in lvPPA were observed across both monolingual and bilingual speakers [13,18,19]. Importantly, bilingual individuals showed variable relative impairment across languages, influenced by dominance, proficiency, and exposure. These findings echo current evidence that bilingual language organisation modulates neurodegenerative decline [12]. In PPA, where linguistic impairment is the primary symptom, such variability demands careful interpretation in clinical contexts.

Logographic impairment adds a further dimension to Chinese PPA profiles, due to both its receptive [35] and expressive [36] implications. Radical-level substitutions, visually confusable errors, and mislocated strokes [8] demonstrate that PPA disrupts orthographic-semantic pathways in ways shaped by logographic complexity. These errors are not directly comparable to alphabetic spelling errors; therefore, they reinforce the need for Chinese-specific writing assessments.

Taken together, the evidence supports a central conclusion, i.e., PPA affects Chinese speakers through mechanisms consistent with universal PPA pathology, yet the manifestation of impairment is filtered through the structural and cognitive demands of tone, compounding morphology, and logographic script. Bilingual experience further shapes how deterioration unfolds across languages.

### 4.2. Why Existing Assessments Fall Short for Chinese Bilingual Individuals with PPA (RQ2)

The reviewed studies also demonstrate that diagnostic tools widely used in PPA research and practice are neither linguistically nor culturally optimised for Chinese-speaking populations. This limitation is most apparent in naming, repetition, and writing assessments.

Naming tasks, including the Boston Naming Test, did not reliably differentiate between semantic impairment and cultural or lexical unfamiliarity [14]. This issue arises because item familiarity, lexical frequency, and semantic categorisation differ considerably between Western and Chinese cultural contexts. Moreover, Mandarin’s extensive homophony complicates the interpretation of naming errors, i.e., failure to retrieve a target word may reflect semantic loss, phonological overlap, or competition among visually or phonologically similar morphemes. These complexities reduce the diagnostic precision of naming tasks unless culturally adapted norms and item sets are available.

Repetition tasks, central to lvPPA diagnosis, present a different problem. Chinese languages, e.g., Mandarin and Cantonese, are tonal languages, and tone errors carry lexical meaning. Yet standard repetition scoring derived from alphabetic languages usually fails to account for tone accuracy. As Tee, Deleon [7] demonstrated, Chinese speakers with PPA may repeat the correct segmental sequence but misproduce tone, as a result, yielding a different lexical item. Western repetition scoring would classify this as correct, potentially masking phonological impairment. However, recommendations for tone-sensitive repetition and connected-speech assessment are based on a small number of studies that directly examined tone production and perception in Mandarin-speaking individuals with PPA, particularly in nfvPPA and lvPPA. While these findings are limited in scope, they consistently highlight tone-segment disruption as a clinically relevant feature warranting further investigation.

Writing assessments are also misaligned with the Chinese linguistic structure. No validated PPA-specific character-writing assessment exists, and Western batteries lack items that probe radical processing, stroke sequencing, or logographic visuoconstruction. Tee, Lorinda Kwan-Chen [8] showed that character-writing errors provide diagnostic insight, yet such measures are absent from most clinical protocols. Equally important to understand is that the evidence supporting character-writing assessment is currently drawn from a limited number of case-based investigations that documented radical- and stroke-level errors in Mandarin speakers with PPA. These findings should, therefore, be interpreted as indicative rather than comprehensive, pointing to a promising but underdeveloped area of assessment.

Finally, bilingual assessment remains underdeveloped. The bilingual individuals in Filley et al. [18] and Weekes [13] were evaluated using tools without bilingual norms or structured assessment of language dominance, proficiency, or usage. Without these metrics, clinicians cannot determine whether cross-language differences reflect pathological decline or baseline bilingual organisation. CALD neuropsychology also warns that applying monolingual norms to bilingual adults inflates impairment rates [15], a risk that is particularly acute in PPA.

### 4.3. What Can We Do to Support Chinese Bilingual Individuals with PPA? (RQ3)

The combined evidence points to several strategies for improving diagnosis, communication support, and clinical care for Chinese-speaking individuals with PPA. These strategies must address both linguistic mechanisms and sociocultural context.

First, assessment protocols should incorporate tone-sensitive repetition tasks, character-based writing assessments, and culturally appropriate naming materials. The diagnostic value of tone production and tone-segment integration in Mandarin nfvPPA and lvPPA [7] suggests that tone should be considered an essential diagnostic variable in Chinese-speaking populations. Similarly, logographic error patterns offer valuable information about orthographic-semantic disruption and should be formally documented using structured character-writing assessments. Developing such tools would represent a substantial advance in Chinese PPA diagnosis.

Also, bilingual assessment requires systematic integration of language history, dominance, proficiency, and language entropy measures. The variability observed across bilingual speakers [13,18] underscores that bilingual decline cannot be interpreted without baseline information. Bilingual PPA assessment should follow frameworks established in multilingual aphasia research, including dual-language sampling and assessment in both dominant and nondominant languages, in which speech–language pathologists play a leading role [37].

Notably, although bilingualism emerged as a relevant factor across several included studies, most available evidence concerns Chinese-English bilinguals, with limited representation of intra-Sinitic bilingualism. As a result, direct comparison of PPA profiles across monolingual Chinese speakers, intra-Sinitic bilinguals, and Chinese-non-tonal bilinguals remains constrained. Nevertheless, the findings suggest that bilingual PPA should be conceptualised along a continuum shaped by tonal overlap, script sharing, and relative language dominance rather than as a single category. These dimensions are likely to modulate cross-language decline, assessment sensitivity, and clinical interpretation, highlighting the need for future research that systematically stratifies bilingual profiles in Chinese-speaking populations.

Sociocultural barriers to help-seeking must be addressed, too. Evidence that Chinese-speaking individuals often delay diagnosis due to stigma or cultural beliefs about ageing [16] indicates that clinician outreach and culturally adapted education resources are necessary. Communication-partner training should be framed within family-centred models of care, as family caregivers often provide primary support [32]. Strategies such as supported conversation, bilingual cueing, and the use of culturally relevant communication routines may help families maintain interactional quality in the presence of progressive language decline. It is worth pointing out that although concepts such as neural reserve in bilingualism and caregiver burden are well established in the broader dementia literature, their relevance here is discussed in relation to the observed language trajectories and family communication challenges reported in the included studies rather than as direct findings.

Clinicians working with Chinese-speaking individuals should adopt culturally responsive approaches that acknowledge the linguistic and cultural diversity of Chinese communities. This includes recognising differences among Mandarin, Cantonese, and other Chinese varieties; understanding the sociocultural dynamics of migration and bilingualism; and tailoring intervention goals to match the client’s linguistic environment and communicative priorities.

These findings highlight the need for linguistically inclusive diagnostic tools, bilingual assessment frameworks, and culturally grounded communication support. They also underscore that PPA research must expand beyond Indo-European languages to reflect the lived experiences of linguistically diverse populations.

### 4.4. Limitations of the Evidence Base

Although the studies included in this review provide valuable insights into PPA in Chinese-speaking populations, several limitations within the evidence base must be acknowledged. The total number of published empirical studies remains small, and many rely on single-case or very small samples. This limits the generalisability of findings and makes it difficult to draw firm conclusions about population-level patterns across PPA variants. Only one study included a sample larger than 50 participants [19], which underscores the need for further large-scale investigations.

Variant representation is uneven. While nfvPPA and lvPPA are represented across multiple studies, svPPA appears only in isolated cases. This imbalance reflects broader trends in the PPA literature but restricts understanding of how semantic decline manifests in Chinese speakers, whose language structure differs markedly from that of Indo-European languages.

Another limitation is the narrow linguistic scope of the current literature. Most included studies examined Mandarin, the standard Chinese. Cantonese, Shanghainese, Hokkien, Teochew, and other Sinitic varieties remain almost entirely unexplored, despite substantial structural differences in tone systems, lexical homophony, and morphology. Given the linguistic diversity of Chinese-speaking populations [6], the current evidence base provides an incomplete picture. The bilingual studies, while valuable, are also limited by inconsistencies in assessment protocols, a lack of bilingual norms, and the absence of language dominance measures. Without these metrics, observations of parallel or asymmetric decline cannot be fully interpreted, therefore making cross-linguistic patterns difficult to analyse systematically.

None of the included studies offered longitudinal data. As a result, the trajectory of tone, semantic, phonological, or orthographic decline in Chinese bilingual PPA remains largely unknown. Cross-sectional data provide important baseline insights, but progression patterns are essential for improving prognosis, communication planning, and intervention design [38].

### 4.5. Future Directions

The limitations of the current evidence base point to several important directions for future research. First, there is an urgent need for the development of Chinese-specific diagnostic tools for PPA. These should include tone-integrated repetition tasks, semantic association tests calibrated for Chinese cultural knowledge, and structured character-writing batteries sensitive to radical and stroke-level errors. Such tools would advance both clinical assessment and research precision.

Because the available evidence is predominantly derived from Mandarin-speaking participants, the conclusions of this review should not be assumed to generalise across all Chinese languages. Linguistic diversity within the Sinitic family represents a critical direction for future research rather than a resolved empirical finding. Therefore, research must also expand beyond the standard Chinese to include other Sinitic varieties. Cantonese, with its nine contrastive tones depending on dialect, presents different cognitive-linguistic demands than Mandarin (typically four tones); similarly, Shanghainese, the lingua franca of Wu, employs a tone-sandhi system (with seven tones) distinct from Mandarin and Cantonese. Exploring PPA across these varieties will deepen understanding of how neurodegeneration interacts with tone systems, morphological typology, and lexical structure.

Third, bilingual PPA requires much more systematic investigation. Future research should include assessments of language dominance, dual-language performance, code-switching patterns, and longitudinal decline across languages. Multilingual PPA research outside Chinese contexts suggests that bilingual trajectories may differ markedly from monolingual ones [39]; therefore, structured investigation in Chinese-English contexts is essential for building clinical guidelines. For example, related bilingual intervention work in acquired aphasia has demonstrated the feasibility of adapting structured verb-retrieval treatments for Mandarin-English bilinguals [40], potentially offering useful design lessons for future PPA-focused intervention development even though acquired aphasia and PPA differ in aetiology and trajectory. Also, as the methodological appraisal (Table 4) highlights the strength of detailed linguistic characterisation across studies, it also underscores the need for larger and longitudinal research to map how tone accuracy, lexical retrieval, semantic knowledge, and logographic writing deteriorate over time. Such studies would support prognostic modelling, inform communication-partner interventions, and guide the timing of AAC introduction and family counselling.

Future research would also benefit from closer integration of linguistically informed assessment with neurobiological markers of disease. Multimodal approaches combining detailed speech–language profiling with structural MRI and molecular imaging techniques, such as amyloid and tau PET, may help refine PPA classification in Chinese and Chinese-English speakers, particularly in cases where language presentation is atypical or variant boundaries are unclear. Additional modalities, including FDG PET and functional imaging, may further elucidate how neurodegenerative pathology interacts with tonal phonology, logographic processing, and bilingual language organisation. Integrating neurobiological and linguistic evidence has the potential to improve diagnostic confidence and advance more precise, person-centred models of PPA across linguistically diverse populations.

There is also a need for translational research examining families’ communication needs, stigma experiences, and help-seeking patterns [41], as wee as the role of AI in enhancing clinician-client-family communication [42]. Understanding how Chinese-speaking families navigate PPA will inform culturally responsive service delivery and support the development of communication-partner training that addresses tone, bilingualism, and sociocultural expectations.

Interdisciplinary collaboration between linguistics, neuropsychology, speech pathology, and cultural health research will be essential for advancing this field. Chinese PPA is inherently multidisciplinary, and progress will rely on integrating diverse expertise to build linguistically inclusive models of neurodegeneration.

## 5. Conclusions

This review synthesised the emerging evidence on primary progressive aphasia in Chinese bilingual speakers to address three questions: what happens to speech and language in this population (RQ1), how well existing diagnostic tools capture these impairments (RQ2), and what we can do to improve clinical care (RQ3). Across the eight included studies, we observed that PPA in Chinese speakers reflects the universal diagnostic features of the syndrome, yet manifests through language-specific pathways shaped by tonal phonology, homophony-driven lexical structure, and the visuoconstructional demands of logographic writing. These features influence not only how impairment presents but how it is detected, understood, and supported.

Our study shows that commonly used Western-derived assessments do not sufficiently capture tone accuracy, character-level writing disruption, or bilingual linguistic organisation. These diagnostic gaps have direct implications for variant classification, care planning, and communication support. We also demonstrate that sociocultural factors, including stigma, family-based care expectations, and bilingual household dynamics, further shape the experience of PPA for Chinese-speaking individuals. Together, these findings highlight why this population cannot be meaningfully assessed or supported using tools and assumptions derived solely from Indo-European language contexts.

From a clinical perspective, the findings of this review suggest several immediately applicable strategies. Assessment of Chinese-speaking individuals with suspected PPA should incorporate tone-sensitive repetition and connected-speech tasks to capture phonological impairment that may not be detected by segment-based measures alone. Evaluation of written language should move beyond alphabetic spelling paradigms to include character-writing tasks that examine radical structure and stroke organisation. For bilingual speakers, systematic documentation of language history, dominance, and daily language use is essential for interpreting differential decline across languages. Furthermore, intervention planning should prioritise culturally responsive communication-partner training that acknowledges family-centred care practices and addresses the functional impact of tone and homophony on everyday communication. Together, these steps provide a practical starting point for clinicians seeking to deliver more linguistically and culturally appropriate care for Chinese and Chinese-English individuals with PPA.

By integrating evidence across linguistics, neuropsychology, CALD health, and bilingualism, this review contributes to a clearer understanding of the mechanisms and clinical challenges unique to Chinese PPA. It also identifies concrete opportunities for progress, i.e., the development of tone-sensitive repetition and character-writing assessments, culturally grounded education and communication-partner training, and bilingual assessment frameworks that reflect real-world language use. Addressing these gaps will lead to more equitable diagnostic pathways and more effective support for Chinese bilingual individuals living with PPA.

In doing so, we move beyond describing deficit patterns to clarifying what must change in clinical practice and research to better serve linguistically diverse populations. As global migration and multilingualism continue to reshape neurodegenerative care, the insights from Chinese PPA are not peripheral; they are rather essential for building a more inclusive science of language, cognition, and neurodegeneration.

## Figures and Tables

**Table 1:** PICO Framework.

Element	Operational Definition
Population	Adults diagnosed with Primary Progressive Aphasia (svPPA, nfvPPA, lvPPA) or FTD with language-led phenotypes, who are bilingual Chinese speakers (Mandarin, Wu, Cantonese, or other Sinitic languages).
Index/Exposure	Language impairment associated with PPA as expressed in bilingual Chinese speakers: tonal phonology, lexical-semantic breakdown, orthographic processing, naming performance, bilingual linguistic profiles, and test performance on culturally adapted or original tools. Includes tone processing, radical and stroke-level writing errors, naming trajectories, and cross-linguistic impairment, etc.
Comparator	No formal comparator required. Implicit comparisons include across PPA variants; cross-linguistic performance (e.g., L1 Chinese vs. L2 English); comparison with normative Chinese test data; comparison of diagnostic tools; and differences between AD vs. FTD/PPA where relevant.
Outcomes	Speech–language characteristics (tone errors, naming accuracy, semantic errors, motor speech features), assessment outcomes, diagnostic clarity/differentiation, bilingual performance patterns, clinically relevant recommendations, and implications for culturally responsive assessment/intervention.

**Table 2:** Inclusion and Exclusion Criteria.

Category	Criteria
Inclusion Criteria	Peer-reviewed empirical studies (case reports, case series, cross-sectional, cohort, diagnostic studies). Participants diagnosed with PPA (any variant), or FTD with language-led phenotype, where Chinese participants with PPA features are described. Participants are bilingual Chinese speakers (Mandarin/Wu/Cantonese/other Sinitic languages). Study reports speech, language, writing, naming, communication, or diagnostic findings relevant to PPA. Published in English. Published after 1 January 2000.
Exclusion Criteria	Studies without any Chinese or Chinese bilingual participants. Studies on dementia without language-led impairment (unless PPA subgroup results are reported separately). No empirical data (e.g., theoretical papers, non-data commentaries). Conference abstracts without full text, or other non-peer-reviewed studies. Studies focusing solely on neuroimaging/genetic pathways without reporting language/communication outcomes. Non-English publications. Publications before 2000.

**Table 3:** Study characteristics.

Study	Design	Diagnosis	Core Speech–Language Findings	Assessment/Diagnostic Tools	Bilingual Implications
Chao, Rosen [17]	Case studies	svPPA with detailed language profiles	Semantic deficits, naming impairment, behavioural changes consistent with svPPA	Clinical neuropsychological battery; qualitative language testing	Diagnostic delay, cultural barriers, and under-recognition of PPA in CALD contexts
Filley, Ramsberger [18]	Single case report	PPA with conduction-type aphasia features	Naming impairment, word-finding pauses, similar patterns across Chinese and English	Boston Naming Test; cross-linguistic observations	Parallel impairment across languages; bilingual assessment relevance
Jebahi, Lai [19]	Case study	lvPPA	Progressive anomia, phonological retrieval deficits, predictors of naming decline	Psycholinguistic naming battery; longitudinal naming tasks	Interaction of bilingualism with naming trajectories and clinical monitoring
Kuo, Tseng [20]	Diagnostic study	nfvPPA	Motor speech impairment; expressive agrammatism; features distinguishing PPA from AD	MoCA, FCSRT, language tasks, ToMT, neuroimaging	Need for culturally adapted diagnostic tools in non-Western contexts
Li, Yu [14]	Diagnostic performance study (cross-sectional)	PPA within mixed neurodegenerative groups	Naming errors, semantic deficits, and patterning on Chinese BNT composite indices	Chinese Boston Naming Test (CP-BNT), composite indices	Lexical/semantic test validity issues in Chinese; implications for PPA diagnosis
Tee, Deleon [7]	Case report	nfvPPA + PSP	Tone production errors, motor speech deficits, and articulatory planning disruptions	Tone identification tasks; language battery across Cantonese/English	Tone-specific impairment and cross-linguistic motor speech issues
Tee, Lorinda Kwan-Chen [8]	Case studies	Mixed PPA variants	Dysgraphia phenotypes: radical substitutions, stroke-order errors, phonologically plausible errors	Chinese writing tasks; writing error analysis	Logographic-specific breakdown; diagnostic utility of Chinese-specific orthographic tasks
Weekes [13]	Narrative review	PPA within ADOD	Cross-linguistic patterns in naming, word-finding, and tone processing	Short Screening Test; cross-linguistic evidence	Culturally valid screening and assessment for multilingual and indigenous Chinese speakers

**Table 4:** Summary of Methodological Quality Appraisal of Included Studies.

Study	Participant Characterisation	Diagnostic Transparency	Data Validity	Reporting Adequacy	Overall Appraisal
Chao, Rosen [17]	Clear	Clear	Adequate	High	Moderate-High
Filley, Ramsberger [18]	Clear	Clear	Adequate	High	Moderate-High
Jebahi, Lai [19]	Clear	Clear	High	High	High
Kuo, Tseng [20]	Clear	Clear	High	High	High
Li, Yu [14]	Clear	Clear	High	High	High
Tee, Deleon [7]	Clear	Clear	Adequate	High	Moderate-High
Tee, Lorinda Kwan-Chen [8]	Clear	Clear	Adequate	High	Moderate-High
Weekes [13]	Clear	Clear	Adequate	High	Moderate-High

Note. Appraisal reflects methodological adequacy relative to study design and research aims, not comparative risk of bias.

**Table 5:** Speech–Language Features and Diagnostic Tool Limitations.

PPA Variant	Core Speech–Language Features in Chinese Contexts	Assessment Tools Used	Diagnostic Limitations
nfvPPA	Impaired tone production and perception; agrammatism; slowed speech; motor speech features	Tone discrimination tasks, fluency and repetition tasks, and connected speech analysis	Standard tools rarely test tonal phonology; Western repetition norms are misaligned with Mandarin prosody; motor speech classification is not calibrated for tone errors
lvPPA	Phonological retrieval deficits; impaired repetition; logographic writing errors (radical substitutions, stroke-order disruptions)	BNT/BNT-M; repetition tasks; orthographic writing tasks	BNT is culturally biased; repetition tasks assume stress-based prosody; no validated logographic writing battery for PPA
svPPA	Semantic degradation; impaired confrontation naming; category-specific deficits; Mandarin semantic association breakdown	Naming tasks; semantic association tests	Semantic categories tested are often culturally mismatched; high homophony in Mandarin complicates lexical frequency interpretation
Logographic impairment	Stroke/structure errors; radical-level confusion; visually similar substitutions	Character-writing tasks; dictation	No PPA-specific writing assessment for Chinese; stroke/radical errors are poorly captured in Western tools
Mixed bilingual profiles	Parallel or asymmetric decline across languages; phonological vs. semantic differences are affected by dominance and usage	Bilingual naming; bilingual repetition; cross-language structural analysis	No bilingual norms; differential impairment hard to interpret; no guidelines for language dominance assessment in PPA

## Data Availability

No new data were created or analyzed in this study. Data sharing is not applicable to this article.

## Abbreviations

The following abbreviations are used in this manuscript:

AAC	Augmentative and Alternative Communication
CALD	Cultural and Linguistic Diversity
lvPPA	The logopenic variant of primary progressive aphasia
nfvPPA	The non-fluent/agrammatic variant of primary progressive aphasia
svPPA	The semantic variant of primary progressive aphasia
PPA	Primary Progressive Aphasia
